# Vitamin D Depletion in Pregnancy Decreases Survival Time, Oxygen Saturation, Lung Weight and Body Weight in Preterm Rat Offspring

**DOI:** 10.1371/journal.pone.0155203

**Published:** 2016-08-29

**Authors:** Sine Lykkedegn, Grith Lykke Sorensen, Signe Sparre Beck-Nielsen, Bartosz Pilecki, Lars Duelund, Niels Marcussen, Henrik Thybo Christesen

**Affiliations:** 1 Hans Christian Andersen Children’s Hospital, Odense University Hospital, Odense, Denmark; 2 Clinical Institute, Faculty of Health Sciences, University of Southern Denmark, Odense, Denmark; 3 Institute of Molecular Medicine, Department of Cancer and Inflammation, Faculty of Health Sciences, University of Southern Denmark, Odense, Denmark; 4 MEMPHYS, University of Southern Denmark, Odense, Denmark; 5 Institute of Pathology, Odense University Hospital, Odense, Denmark; Xavier Bichat Medical School, INSERM-CNRS - Université Paris Diderot, FRANCE

## Abstract

Animal studies suggest a role of vitamin D in fetal lung development although not studied in preterm animals. We tested the hypothesis that vitamin D depletion aggravates respiratory insufficiency in preterm rat offspring. Furthermore, the effects of vitamin D depletion on growth and lung surfactant were investigated. Female Sprague-Dawley rats were randomly assigned low vitamin D (VD_L_) or control diet before mating and followed with serum 25-hydroxyvitamin D (s-25(OH)D) determinations. After cesarean section at gestational day 19 (E19) or day 22 (E22), placental weight, birth weight, crown-rump-length (CRL), oxygenation (SaO_2_) at 30 min and survival time were recorded. The pup lungs were analyzed for phospholipid levels, surfactant protein A-D mRNA and the expression of the vitamin D receptor (VDR). S-25(OH)D was significantly lower in the VD_L_ group at cesarean section (12 vs. 30nmol/L, p<0.0001). Compared to the controls, E19 VD_L_ pups had lower birth weight (2.13 vs. 2.29g, p<0.001), lung weight (0.09 vs. 0.10g, p = 0.002), SaO_2_ *(*54% vs. 69%, p = 0.002) as well as reduced survival time (0.50 vs. 1.25h, p<0.0001). At E22, the VD_L_-induced pulmonary differences were leveled out, but VD_L_ pups had lower CRL (4.0 vs. 4.5cm, p<0.0001). The phospholipid levels and the surfactant protein mRNA expression did not differ between the dietary groups. In conclusion, Vitamin D depletion led to lower oxygenation and reduced survival time in the preterm offspring, associated with reduced lung weight and birth weight. Further studies of vitamin D depletion in respiratory insufficiency in preterm neonates are warranted.

## Introduction

At birth, adequate lung development and growth is the key to successful adaptation to extrauterine life [1]. Thus, appropriate lung function depends on both proper development of the lung structure as well as sufficient surfactant synthesis and secretion [1, 2]. In preterm neonates inadequate lung maturation leads to respiratory distress syndrome (RDS) characterized by structural immaturity and inadequate synthesis and secretion of surfactant [2]. Antenatal corticosteroids [3, 4], intratracheal surfactant [5] and nasal continuous positive airway pressure (nCPAP) or mechanical ventilation [6, 7] are important in modern treatment of RDS. Yet, respiratory insufficiency due to lung immaturity is still a major cause of mortality in extremely preterm neonates [6], and research in other treatment modalities are needed.

Placenta plays a vital role in normal fetal development, and fetal or neonatal disorders may be a result of placental insufficiency. Hypovitaminosis D, most commonly defined as s-25(OH)D levels below 50 nmol/L, is frequent in both pregnant women and preterm neonates [8–13]. Some human studies have shown an association between low s-25(OH)D levels during pregnancy and reduced placental development and weight [14–16], while others have not [17, 18]. Both placental weight and placental weight/birth weight (PW/BW) ratio have been used to describe the growth conditions for the fetus [19–22]. In humans, a high PW/BW ratio has been associated with RDS, low apgar scores and increased risk of admission to the neonatal intensive care unit [21, 23]. Only one human study [17] has approached the association between vitamin D and PW/BW ratio, however, without positive findings.

The impact of calcitriol (1,25(OH)_2_D), the active metabolite of vitamin D) on early lung development and maturation as well as on development of lung diseases in early life is an emerging field of research. A recent human study found an association between s-25(OH)D levels below 30 nmol/L and increased oxygenation requirement and greater need of assisted ventilation in preterm neonates [24]. Yet, the human evidence on the impact of 1,25(OH)_2_D on lung development and maturation remains sparse. In contrary, both animal and laboratory studies, primary based on vitamin D enrichment, have provided detailed insights into the mechanisms through which 1,25(OH)_2_D stimulates the development and maturation of the lung [3]. *In vivo* studies in mice [25] and rats [26, 27] have shown that vitamin D deficiency during pregnancy causes alterations in lung growth and structure in term offspring. *In vitro* studies of cells from fetal rat lung explants have shown an impact of vitamin D on the embryogenesis and cellular growth and differentiation, including surfactant synthesis and secretion [28–35]. Laboratory studies on human pulmonary adenocarcinoma-derived cell lines support these findings [34, 36, 37]. However, *in vivo* studies of the role of vitamin D in lung development in preterm offspring have not been performed.

We therefore tested the hypothesis that vitamin D depletion in pregnancy does not aggravate respiratory insufficiency in the preterm rat offspring.

## Materials and Methods

### Animals

Sprague-Dawley rat dams were purchased from Taconic Biosciences (Denmark) and housed in a facility without windows and light without UV-B radiation. All fluorescence lamps were shatterproof and had built-in UV-filters (Phillips MASTER TL-D Secura 58W/830 (150cm)). Room air was kept at 21–24 degrees Celsius and the animals were exposed to day-night cycles alternating every 12 hours. The animals (n = 34) were randomly assigned to two different dietary groups; a low vitamin D (VD_L_) diet group (n = 18) (<5 IU/kg cholecalciferol, purified vitamin D_3_ deficient diet, Art No E15312-24*)* and a control diet group (n = 16) (1500 IU/kg cholecalciferol, purified control diet, Art No E15000-04*)*. Both diets were designed for maintenance, were identical in terms of all other components including calcium and phosphorus and were obtained from Ssniff, Soest, Germany. Adult ten-week-old females were fed *ad libitum* for five weeks with the assigned diet before mating with adult males and continued throughout the pregnancy. The males were maintained on a standard rat chow (600 IU/kg cholecalciferol, Art No #1320, Altromin Spezialfutter, Lage, Germany). The pregnant females from each dietary group were randomly assigned to either the E19 or E22 subgroup immediately after the appearance of vaginal plug (gestational day 0 (E0)). Blood samples were collected from the tail vein before mating and after cesarean section before euthanizing. To observe the welfare, the weight of the pregnant rats was followed throughout the study. In a supplementary study, a whole-body dual-energy x-ray absorptiometry (DXA) scan of some of the pups from each dietary group was performed to describe the bone mineral content. The Danish National Animal Experiments Inspectorate approved the study (permit number: 2012-15-2934-00243).

### Cesarean section and survival

On gestational day 19 (E19) or 22 (E22)—roughly equivalent to human gestational week 24 and term, pregnant mothers were anesthetized with isoflurane inhalation and the pups were recovered by cesarean section. Maternal oxygen saturation (SaO_2_) was monitored during the procedure and all efforts were made to minimize suffering. After removal of the pups, serum samples from the mothers were collected by intracardiac puncture followed by euthanization with pentobarbital sodium. Newborn pups were immediately wiped dry with a cloth and placed on a heating pad. After the cesarean section, the placenta and pups were weighed and the pup crown-rump length (CRL) was assessed within 30 minutes after birth. SaO_2_ was measured at 30 minutes after birth, and the pups were randomly, selected to be sacrificed immediately hereafter or kept with foster mother rats. The lungs from the euthanized pups were weighed and dissected for qPCR (quantitative real-time PCR) analysis, immunohistochemical analysis and phospholipid analysis. An investigator blinded to the dietary group monitored the survival of the non-sacrificed pups with a frequency of 15 minutes for the first two hours. The pups were euthanized if no movements, low heart rate and undetectable low SaO_2_ were observed. To observe the welfare and describe the survival time of the E22 pups, the blinded investigator registered daily weight, the ability to suckle and the activity level the first seven days after birth. Our human endpoints were observed distress and/or affected welfare described as weight loss > 20% within the first two days of life, inactivity and/or ruffled fur. In case of doubt, a veterinarian examined the pups.

### Oxygen saturation

SaO_2_ was measured by using Nonin Pulse Oximeter^®^ on the rats and pups in room air. The sensor was attached to the precordial site, and the animals were placed in a prone position to maintain a good attachment of the sensor. During the measurements, the pups were kept on a heating pad to avoid hypothermia. The readings of SaO_2_ were accepted as valid when the simultaneously monitored heart rate was stable. Each measurement was finished within 10–15 seconds to avoid desaturation secondary to prolonged measurement. For each animal, three to five measurements were taken within 2 minutes and the highest value of SaO_2_ was used for analysis.

### Serum 25(OH)D, calcium and phosphorus levels

Serum samples were centrifuged and kept frozen at -20°C until analysis. All analyses were made after the study was finished. While the levels of s-25(OH)D were measured by liquid chromatography-tandem mass spectrometry (LC-MS/MS) [38], serum phosphorus and calcium levels were measured using a Roche Cobas 8000 Autoanalyzer Spectrophotometric (Roche Diagnostics^®^).

### qPCR analysis

To quantify mRNA expression of the surfactant protein A-D genes (*sftpa*, *sftpb*, *sftpc*, *sftpd)*, total RNA was extracted from homogenized lung tissue using TRIzol reagent (Life Technologies) according to the manufacturer’s instructions. Two μg RNA were used for cDNA production. Reverse transcription was performed using M-MLV Reverse Transcriptase (Sigma), and the reaction product was diluted to a final concentration of 10 ng/μl. Real-time PCR was performed in duplicates using the TaqMan Universal PCR Master Mix and TaqMan Gene Expression Assays specific for the given gene. The assay kits used were as follows: *Gapdh*, Rn01775763_g1; *Sftpa1*, Rn00824545_m1; *Sftpb*, Rn00684778_m1; *Sftpc*, Rn00569225_m1; *Sftpd*, Rn00563557_m1. The results were calculated using the 2^ΔΔCt^ method.

### Phospholipid levels

Isolated lungs were snap-frozen in liquid nitrogen and stored at -80°C until analysis. Lipid extraction was performed using a MTBE/methanol/ammonium acetate protocol [39]. Afterwards, the phospholipid concentration was determined in duplicates using a modification of the Bartlett method [40]. Briefly, 0.65 ml 70% perchloric acid was added to the dried samples followed by heating at 190°C for 60 minutes. Thereafter, the cooled samples were mixed with 3.3 ml water, 0.5 ml 2.5% ammonium molybdate and 0.5 ml 10% ascorbic acid followed by incubation at 100°C for 10 minutes. Finally, 250 μl from each cooled sample was transferred to a 96-well microtiter plate and absorbance was read at 800nm.

### Immunohistochemical detection of VDR

Isolated lungs were immersed in 4% paraformaldehyde at room temperature for 24 hours fixation, processed, embedded in paraffin wax and cut into sections of 1.5 μm thickness. Before nuclear staining with Mayer’s hematoxylin, the sections were pretreated with MBO/TEG for 15 minutes followed by D-6 monoclonal antibody against VDR (dilution 1:500, Santa Cruz Biotechnologies cat. no. sc-13133). The sections were scanned, and microscopic analysis was performed using newCAST software (Visiopharm, Hoersholm, Denmark). Each section was analyzed according to previously described procedures [41] by an investigator blinded to the dietary group as well as the gestational age of the pups. The expression of VDR in the lung was estimated as the density of VDR-positive alveolar type II cells (cells/μm^2^ lung tissue).

### Statistics

Shapiro-Wilks test revealed non-normal distribution of s-25(OH)D, maternal SaO_2_, CRL and lung weight. Between-group comparisons were made using two-way ANOVA and two-tailed unpaired *t-*tests, or non-parametric Mann-Whitney U test when appropriate. Survival analysis was performed using log-rank Mantel-Cox test. Normally distributed data were presented as mean ± SEM and non-normally distributed data as median and range. Spearman’s correlation was used to study within group correlations between SaO_2_ and LW, BW, LW/BW ratio and PW/BW ratio.

A power calculation assuming an E19 SaO_2_ SD of 10, two-sided alpha 0.05 and beta 0.20, showed a need of 34 pups to detect a true difference in SaO_2_ of 10 between groups. As we only expected to be able to measure SaO_2_ in one pup per litter, mother rat n = 34 were chosen.

Due to a substantial overlap in s-25(OH)D levels between the two dietary groups, we further performed a *post hoc* analysis. In this analysis associations between maternel s-25(OH)D at cesarean section and the measured phenotypical outcomes (SaO_2_, PW, BW, LW, LW/BW ratio, PW/BW ratio and CRL) were analyzed using linear uni- and multivariate regression models disregarding of initial dietary group. Moreover, we also performed a *post hoc* survival analysis comparing pups of mothers with s-25(OH)D <25nmol/L to pups of mothers with s-25(OH)D ≥25 nmol/L disregarding of initial dietary group.

A p-value <0.05 was considered statistically significant; p = 0.05–0.10 was considered a trend. Data were analyzed using the Prism software package (version 6.0, GraphPad) and STATA software, version 13.1 (StataCorp, College Station, TX, USA).

## Results

### Maternal data

Before mating, the median s-25(OH)D in the VD_L_ group was lower compared to the controls, but no significant differences in s-total calcium or s-phosphorus were found between the two dietary groups, Table 1. However, the finding of lower s-25(OH)D values in the VD_L_ group only reached significance in the E22 subgroup, S1 Table.

**Table 1:** Maternal data.

	VD_L_ group (n = 18)	Controls (n = 16)	p-value
S-25(OH)D before mating (nmol/L) *	43 (24–130)	64 (42–118)	0.009
S-25(OH)D after CS (nmol/L) **	12 (8–27)	30 (17–137)	< 0.0001
S-total calcium (nmol/L) before mating *	2.64 ± 0.03	2.62 ± 0.02	0.547
S-total calcium (nmol/L) after CS**	2.72 ± 0.04	2.92 ± 0.07	0.018
S-phosphorus (nmol/L) before mating *	2.61 ± 0.08	2.84 ± 0.15	0.153
S-phosphorus (nmol/L) after CS**	1.84 ± 0.05	2.06 ± 0.08	0.028
Weight (g) at arrival to the laboratory	262 ± 3	263 ± 4	0.778
Weight (g) before CS	386 ± 9	386 ± 13	0.967
Maternal weight gain (g)	124 ± 9	122 ±13	0.896
Duration of maternal anesthesia (min)	7.59 ± 0.40	6.58 ± 0.60	0.158
Maternal SaO_2_ (%)	100 (98–100)	99 (95–100)	0.292
Litter size (n)	13 ± 0.8	12 ± 1.3	0.345

*Tail vein.

**Intracardial puncture. Normal distributed data presented as mean ± SEM.

Non-normal distributed data presented as median (range). Abbreviations: S-25(OH)D: serum 25-hydroxyvitamin D, CS: cesarean section; SaO_2_: oxygen saturation.

At cesarean section, both s-25(OH)D levels and s-phosphorus levels had decreased in both dietary groups and s-total calcium levels increased, Table 1. At time of cesarean section, s-25(OH)D was significantly lower in the VD_L_ animals compared to controls both at E19 and at E22, S1 Table. In the E19 control subgroup, the s-25(OH)D had a median of 25 nmol/L, e.g. an unintended moderate s-25(OH)D deficiency level, whereas the median in the E22 subgroup was 53 nmol/L, e.g. a normal s-25(OH)D level. S-total calcium and s-phosphorus became significantly lower in the VD_L_ animals compared to controls at cesarean section in the E19 subgroup, but not at E22, S1 Table. Both s-total calcium and s-phosphorus were within the laboratory reference range for adult rodents at all times.

All mother rats achieved a weight gain during the experiment with no significant difference in gestational weight gain between the groups. At the cesarean section, no differences were observed in maternal SaO_2_, the duration of maternal anesthesia or in the number of pups in each litter.

### Offspring data

Half of the animals from each dietary group had cesarean section at E19, n = 65 (VD_L_) vs. n = 56 (controls): the other half at E22, n = 75 (VD_L_) vs. n = 49 (controls). When comparing preterm and term offspring in general, a significant difference was observed in all measurements (lung, birth and placental weights and CRL) as the results of maturation, Table 2.

**Table 2:** Offspring data.

	E19 (n = 121)			E22 (n = 124)			p-value (E19 *vs* E22)	
	VD_L_ group (n = 65)	Control group (n = 56)	p-value	VD_L_ group (n = 75)	Control group (n = 49)	p-value	VD_L_ group	Control group
PW (g)	0.58 ± 0.01	0.58 ± 0.02	0.671	0.71 ± 0.01	0.76 ± 0.03	0.074	<0.001	<0.001
BW(g)	2.13 ± 0.03	2.29 ± 0.03	<0.001	5.21 ± 0.08	5.42 ± 0.18	0.245	<0.001	<0.001
LW(g)	0.09 ± 0.001	0.10 ± 0.002	0.002	0.15 ± 0.003	0.15 ± 0.004	0.543	<0.001	<0.001
CRL (cm)	2.8 (2.2–3.2)	2.9 (2.6–3.1)	0.072	4.0 (3.5–4.5)	4.5 (3.9–5.0)	<0.001	<0.001	<0.001
PW/BW ratio	0.28 ± 0.006	0.25 ± 0.01	0.006	0.14 ± 0.003	0.14 ± 0.003	0.314	<0.001	<0.001
LW/BW ratio	0.04 ± 0.001	0.04 ± 0.001	0.011	0.03 ± 0.008	0.03 ± 0.001	0.060	<0.001	<0.001

Normal distributed data presented as mean ± SEM. Non-normal distributed data presented as median (range). Abbreviations: CRL; Crown-Rump Length, LW; lung weight, BW; birth weight, PW; placenta weight.

When comparing the two dietary groups at E19 or E22, respectively, there was no difference between the weights of placenta, but a significant difference in PW/BW ratio was observed at E19, giving a higher ratio in the VD_L_ pups compared to control pups due to lower body weight. At E19, birth weight, lung weight and lung weight/birth weight ratio (LW/BW ratio) were significantly lower in the pups born of VD_L_ mothers than in the controls. While only insignificant difference in CRL was observed between the two dietary groups at E19, pups born at E22 of VD_L_ mothers were significantly shorter than controls. Potential alterations in bone mineral content were not detectable using whole-body DXA scans (data not shown). All E22 weight measurements showed no difference between the dietary groups.

**Fig 1:**
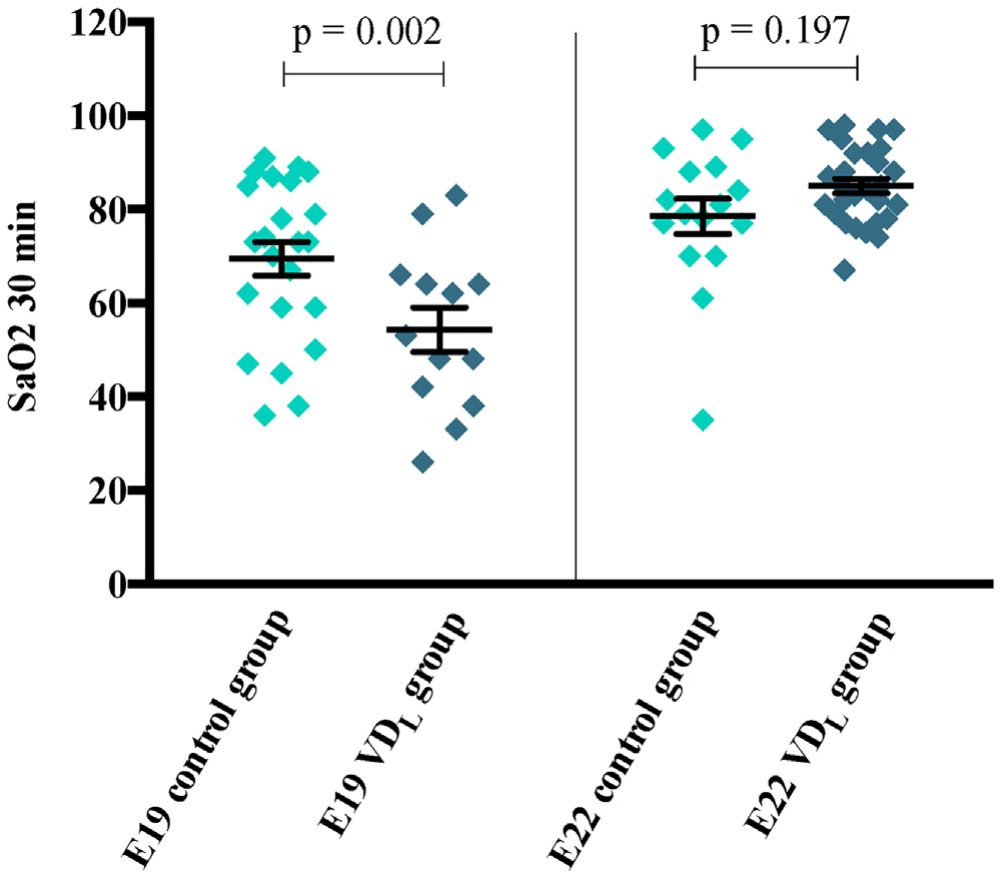
SaO_2_ measured in rat pups 30 minutes after birth. SaO_2_ was measured 30 minutes after birth at E19 (VD_L_ group (n = 13); control group (n = 23)) and E22 (VD_L_ group (n = 28); control group (n = 16)). Within 2 minutes 3–5 measurements were made and the highest value of SaO_2_ was used for analysis. Each measurement was finished within 10–15 seconds to avoid desaturation secondary to prolonged measurement.

Pups born at E19 had significantly (p < 0.001) lower 30 min SaO_2_ values than pups born at E22 (Fig 1). At E19, the pups of VD_L_ mothers further had significantly (p = 0.002) lower SaO_2_ values than controls, whereas no significant difference was observed between the dietary groups at E22.

**Fig 2:**
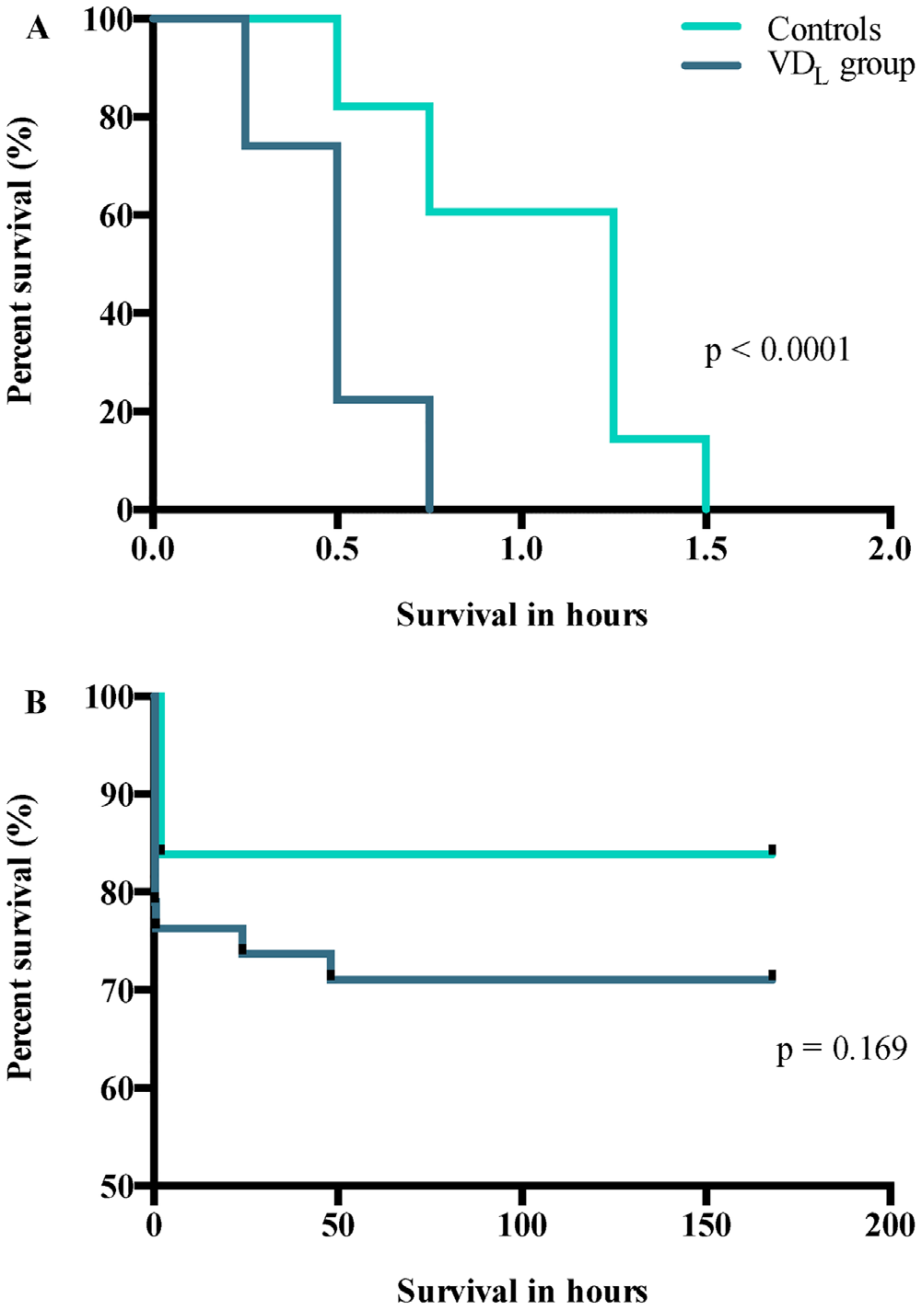
Survival-rate in hours after birth. Kaplan-Meier survival analysis of both A) E19 (VD_L_ group (n = 58); control group (n = 28)) and B) E22 (VD_L_ group (n = 38); control group (n = 31)) pups. Comparison of survival curves using log-rank (Mantel-Cox) test.

At E19, the survival-rate was significantly (p < 0.0001) reduced in the VD_L_ pups compared to controls and none of the pups survived more than 1.5 hours after birth (Fig 2). During the first 24 hours the majority of the E22 pups survived, leading to a 24h survival time of 74% in the VD_L_ group and 84% in the control group. Further 3% of the pups in the VD_L_ group died within day 2, after which all pups survived, leading to no significant difference in the long-term survival time between the groups born at E22 (Fig 2).

Results from correlation analysis are presented in Table 3. In the E19 VD_L_ pups, we found a significant positive correlation between SaO_2_ and birth weight and a trend towards a negative correlation between PW/BW ratio and SaO_2_, but no correlation between lung weight and SaO_2_. In E19 control pups, a significant negative correlation between LW/BW ratio and SaO_2_ was observed.

**Table 3:** Correlations between saturation and lung weight, birth weight, LW/BW ratio and PW/BW ratio.

	SaO_2_ E19 pups				SaO_2_ E22 pups			
	VD_L_ group		Control group		VD_L_ group		Control group	
	r	p-value	r	p-value	r	p-value	r	p-value
**LW**	-0.05	0.864	0.35	0.349	0.08	0.663	0.12	0.655
**BW**	0.63	0.001	-0.18	0.558	-0.26	0.182	0.13	0.625
**LW/BW ratio**	0.09	0.782	-0.53	0.009	0.23	0.227	-0.08	0.763
**PW/BW ratio**	-0.55	0.051	-0.37	0.080	0.34	0.076	0.04	0.892

r = Spearman’s coefficient. Abbreviations: LW; lung weight, BW; birth weight, PW; placenta weight.

### Pulmonary surfactant protein expression, phospholipids and VDR levels

**Fig 3:**
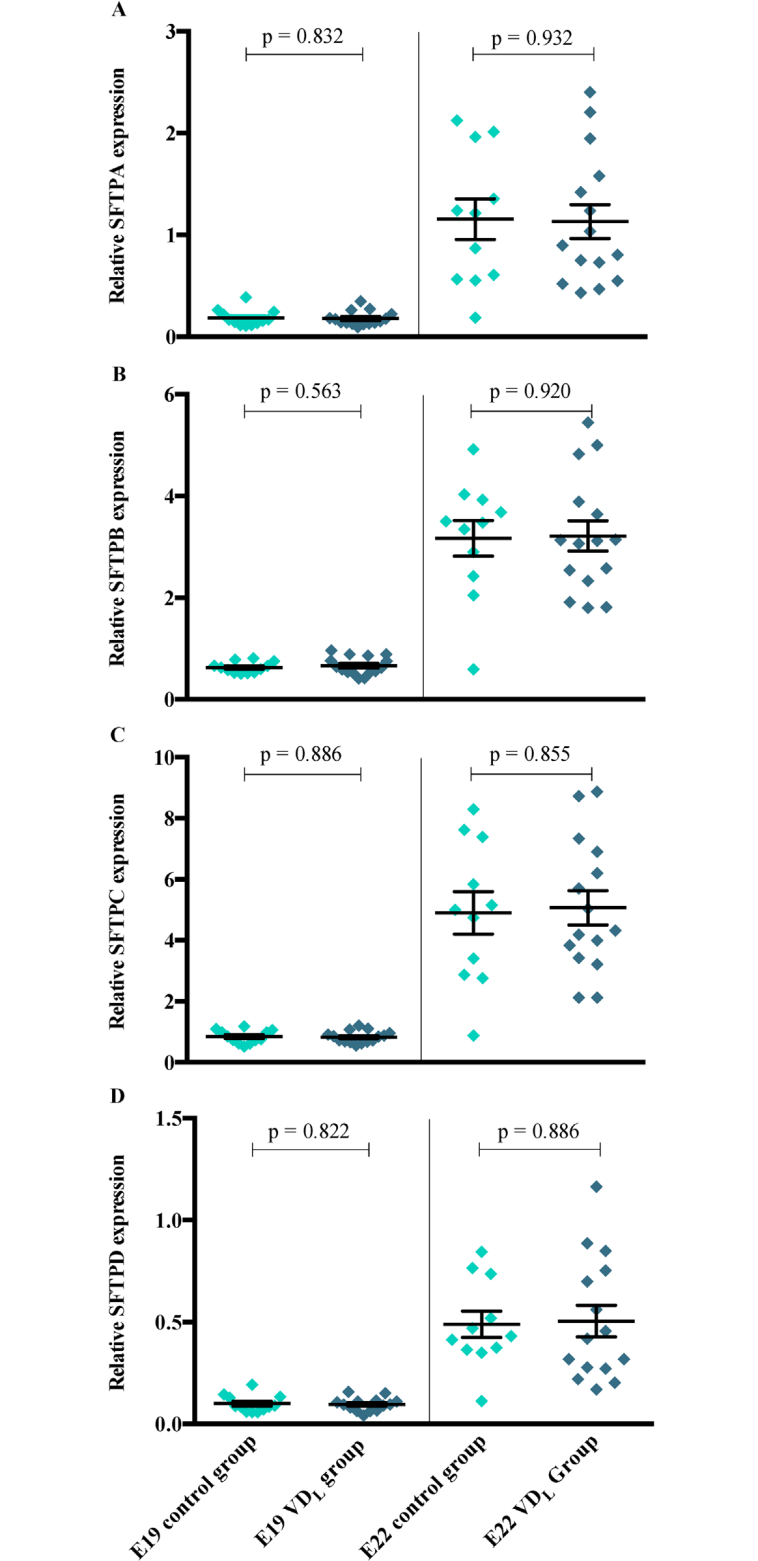
Effects of vitamin D on mRNA levels of surfactant proteins. Quantitative real-time PCR analysis of surfactant protein A-D mRNA transcripts in fetal rat lung at E19, VD_L_ group (n = 15); control group (n = 12) and at E22, VD_L_ group (n = 15); control group (n = 11). Data were normalized against GAPDH. Results were calculated as mean ± SEM values.

**Fig 4:**
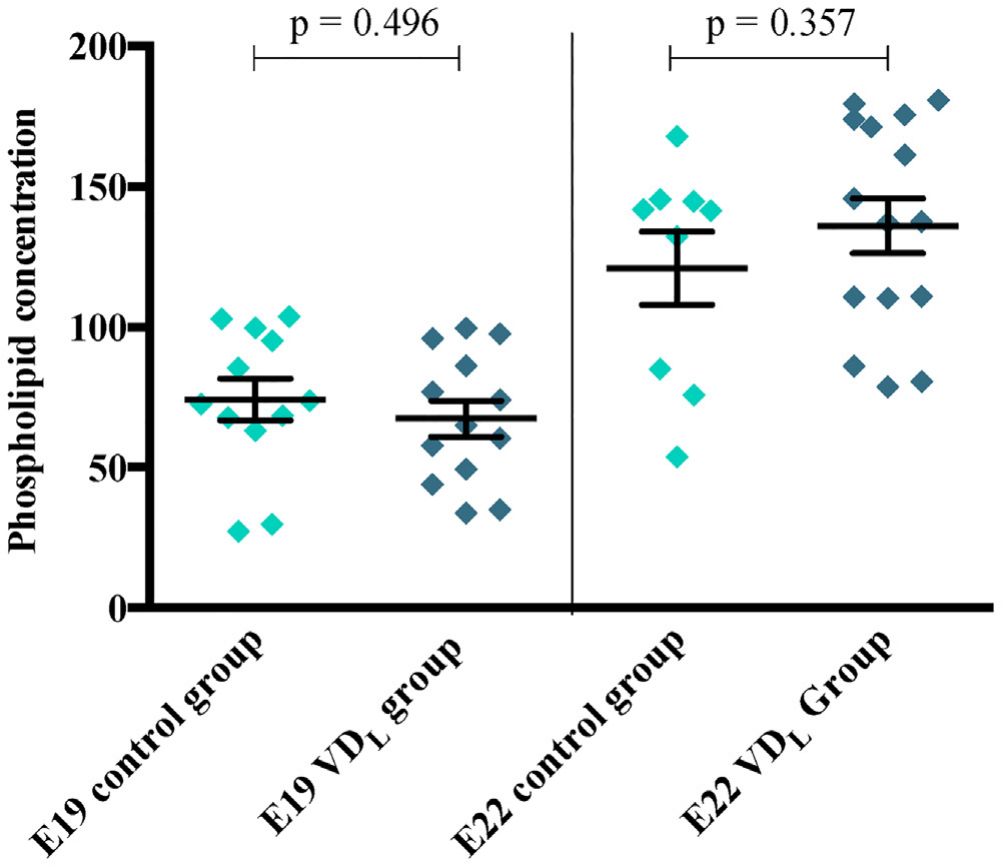
Effects of vitamin D on phospholipid levels. Phospholipid levels in fetal rat lung at E19, VD_L_ group (n = 13); control group (n = 12) and at E22, VD_L_ group (n = 15); control group (n = 9). Phospholipid levels were determined using a modification of the Bartlett method followed by absorbance read at 800nm.

The qPCR analysis showed a significantly lower surfactant protein A-D mRNA levels (Fig 3) in lung tissue obtained from pups born at E19 compared to E22. The concentration of pulmonary phospholipids was also significantly lower in the E19 pups compared to the E22 pups (Fig 4). No significant differences in surfactant protein mRNA expression or phospholipid concentrations were observed when comparing the two dietary groups at either E19 or E22.

**Fig 5:**
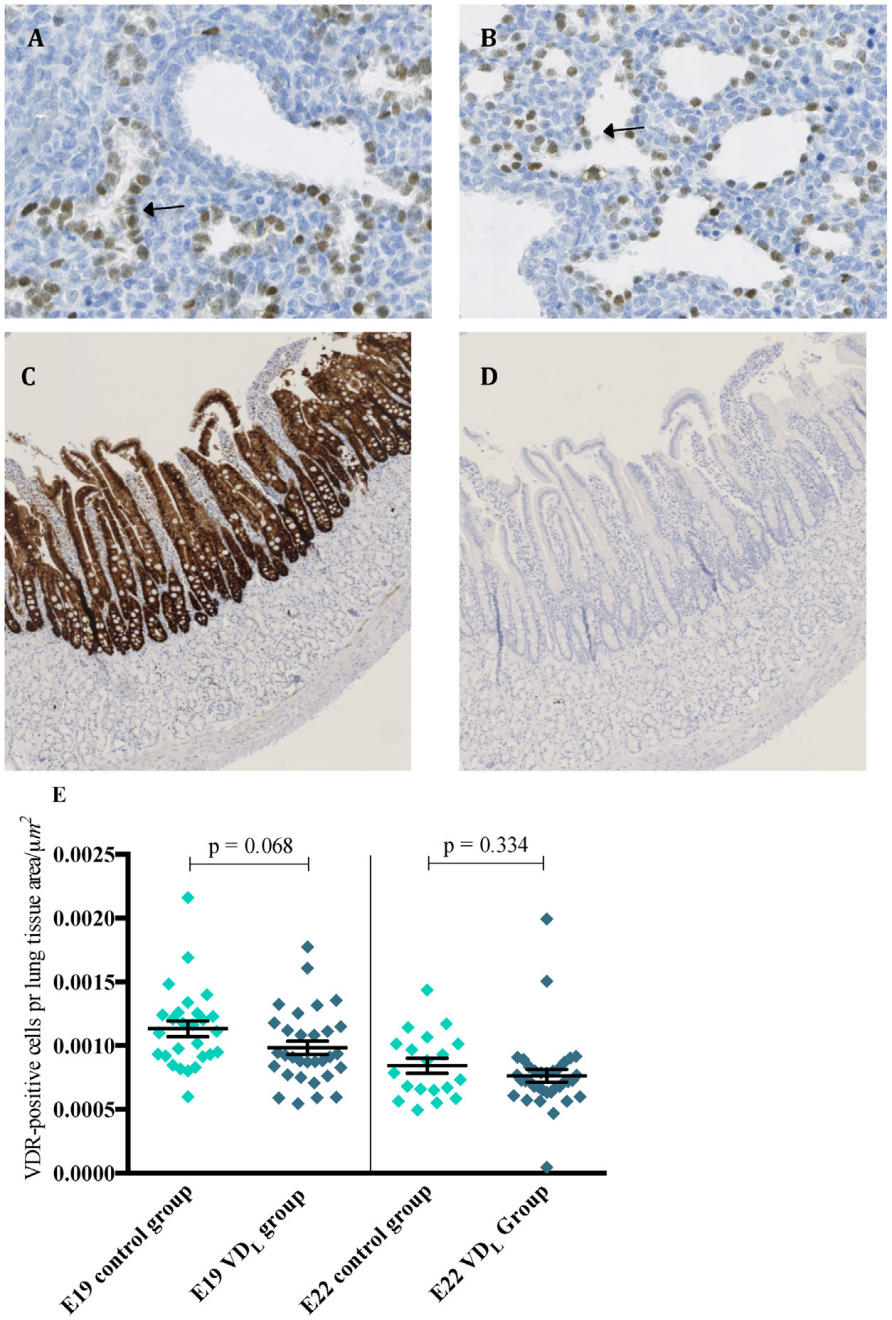
Immunohistochemical analysis of VDR in fetal rat lung. Immunohistochemistry was performed on lung tissues from each dietary group at both E19 (VD_L_ group (n = 31); control group (n = 27)) and E22 (VD_L_ group (n = 35); control group (n = 19)) using the monoclonal D-6 antibody and counterstained with Mayer’s hematoxylin. Representative images of lung tissue from E19 (A) and E22 (B) (Magnification x28). Arrows indicate positive ATII cells. Positive (C) and negative (D) control sections with omission of the monoclonal D-6 antibody in the duodenum (Magnification x5). The expression of VDR in the lung was evaluated as the density of positive ATII cells (cells/μm^2^) (E).

Immunohistochemical analysis showed that VDR was expressed in the ATII cells during the last days of gestation in both dietary groups. The density of VDR-positive ATII cells in lung tissue in both E19 dietary groups was significantly (p < 0.001) higher compared to E22. At E19, a trend (p = 0.068, Fig 5) towards lower levels of VDR was observed in the VD_L_ group compared to controls. The expression of VDR in both E19 groups was significantly higher compared to E22 whereas no difference in VDR expression was found between the dietary groups at E22.

### S-25(OH)D associations with pulmonary outcomes and survival

Due to a substantial overlap in maternal s-25(OH)D between the two dietary groups at cesarean section, *post hoc* analyses on the association between s-25(OH)D and outcomes was performed for all animals disregarding initial dietary group, Table 4.

**Table 4:** Uni- and multivariate linear regression models of the association between maternal s-25(OH)D at cesarean section and SaO_2_, PW, BW, LW, LW/BW ratio, PW/BW ratio and CRL.

	Univariate analyses				Adjusted analyses			
	s-25(OH)D at cesarean section				s-25(OH)D at cesarean section			
	E19		E22		E19		E22	
	β	p-value	β	p-value	β	p-value	β	p-value
**SaO**_2_*	1.87	<0.001	0.62	0.090	2.39	<0.001	1.13	0.113
BW**	0.01	0.001	0.03	<0.001	0.01	0.002	0.03	<0.001
PW**	-0.00	0.334	0.01	<0.001	-0.00	0.100	0.01	<0.001
LW**	0.00	<0.001	-0.00	0.056	0.00	<0.001	-0.00	0.183
PW/BW**	-0.00	0.001	0.00	<0.001	-0.00	<0.001	-0.00	0.302
LW/BW**	0.00	0.006	-0.00	<0.001	0.00	0.006	-0.00	<0.001
CRL**	0.00	0.168	-0.01	<0.001	-0.00	0.867	0.01	<0.001

All animals disregarding initial dietary group were included in the analyses. Adjusted for:

* Maternal duration of anesthesia,

** Littersize and maternal weight.

Abbreviations: *β*: β-coefficient, LW; lung weight, BW; birth weight, PW; placenta weight.

At E19, s-25(OH)D was positively associated with SaO_2_, birth weight, lung weight and LW/BW ratio, and negatively associated with PW/BW ratio.

At E22, maternal s-25(OH)D was positively associated with CRL, birth weight, placental weight and PW/BW ratio and negatively associated with LW/BW ratio.

Adjustment for maternal weight and littersize did not change the associations at E19. But at E22 only birth weight, placental weight and CRL remained positive associated with s-25(OH)

**Fig 6:**
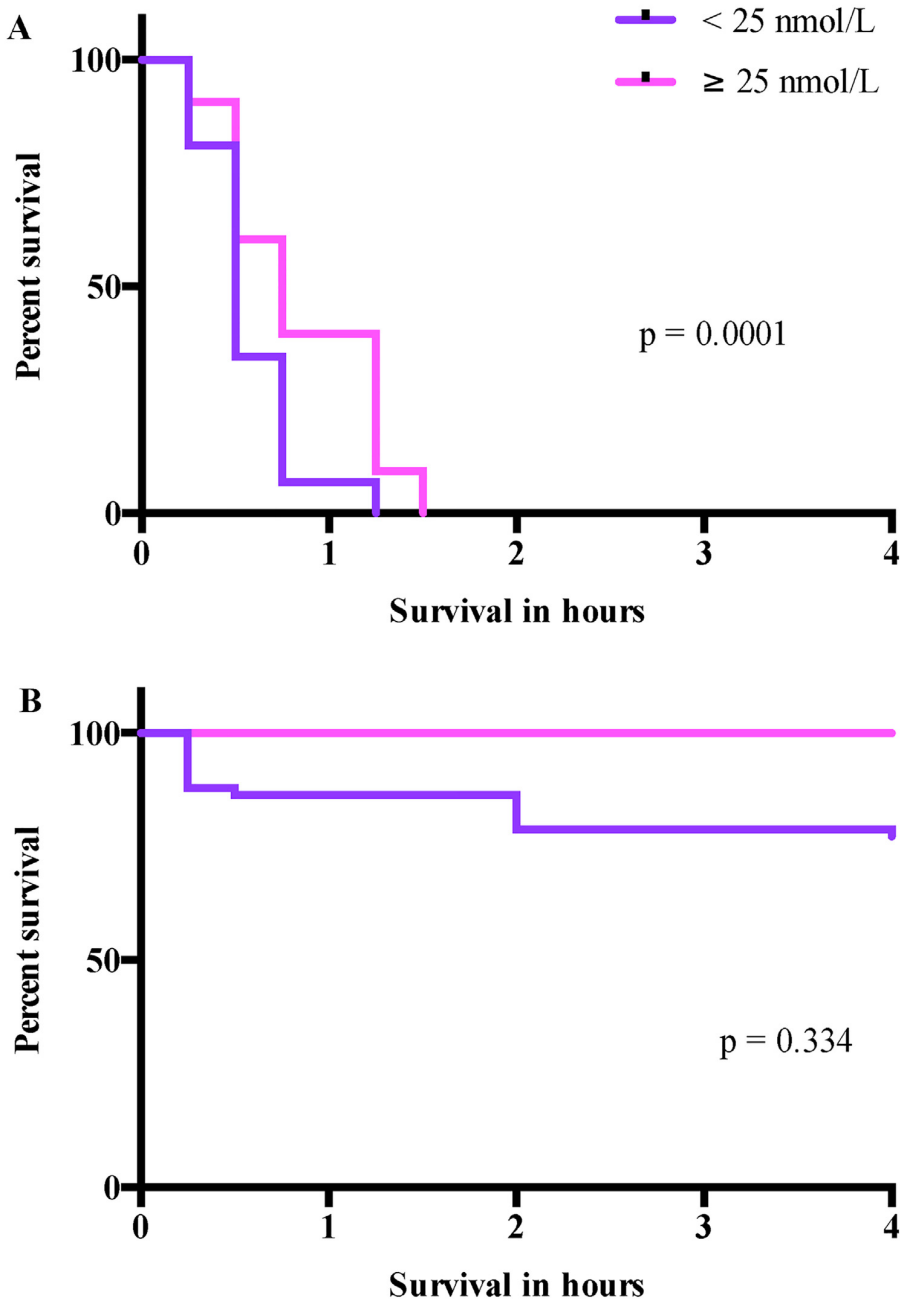
Survival-rate of pups from mothers stratified by s-25(OH)D <25 nmol/L. Kaplan-Meier survival analysis of both A) E19 (s-25(OH)D <25 nmol/L (n = 58); ≥25 nmol/L (n = 43)) and B) E22 (s-25(OH)D <25 nmol/L (n = 58); ≥25 nmol/L (n = 28)) pups. Comparison of survival curves using log-rank (Mantel-Cox) test.

Moreover, a survival analysis comparing offspring of mothers with s-25(OH)D levels <25nmol/L at cesarean section to offspring of mothers with s-25(OH)D levels ≥25 nmol/L was performed (Fig 6). While the survival-rate was significantly (p = 0.0001) lower in the E19 pups of mothers with s-25(OH)D <25 nmol/L compared to s-25(OH)D ≥25 nmol/L, there was no significant difference between the pups of mothers with s-25(OH)D <25 nmol/L compared to s-25(OH)D ≥25 nmol/L at E22.

## Discussion

We found that premature rat offspring from mothers with severe vitamin D deficiency had reduced oxygenation and survival-rate compared to offspring of mothers with moderate vitamin D deficiency. Potential explanatory findings were significantly lower lung weight, birth weight, LW/BW ratio higher PW/BW ratio and lower s-total calcium in the premature VD_L_ pups, whereas no reduction in the expression of surfactant protein A-D mRNA, pulmonary phospholipid concentration, or VDR expression, was found. Furthermore, birth weight and PW/BW ratio correlated significantly to SaO_2_ levels at 30 min in E19 VD_L_ pups. The VD_L_-induced pulmonary outcomes were only detectable in the premature pups and not in fully matured pups, but were supported by direct multivariate associations between s-25(OH)D levels and lung weight and SaO_2_ and decreased survival with s-25(OH)D deficiency in the premature.

Our obtained results support that 25(OH)D is implicated in early fetal growth but are in contrast to the previously described positive impact of vitamin D on the synthesis and secretion of surfactant proteins and phospholipids [3]. We used an animal model of vitamin D deficiency during pregnancy, while most others either studied the *in vitro* synthesis of surfactant by incubating ATII cells with 1,25(OH)_2_D [29, 30, 33], or *in vivo* by vitamin D enrichment before sacrifice [26, 27]. Our model was designed to resemble the clinical setting of hypovitaminosis D in humans and we achieved a s-25(OH)D level of 12 nmol/L in the VD_L_ mothers at cesarean section, which corresponds to severe vitamin D deficiency. In normal pregnant rats, levels of s-25(OH)D gradually decrease during the last period of gestation [42], as did in our control animals, which explains the substantial overlap in low s-25(OH)D between dietary groups. Our negative results on VD_L_-mediated surfactant protein A-D mRNA expression and pulmonary phospholipid levels may be attributed to failure of remaining s-25(OH)D concentrations >50 nmol/L in the E19 control group. Moreover, pups for lung studies were randomly chosen within the dietary groups, which had overlapping maternal s-25(OH)D concentration ranges.

In rats, the synthesis of surfactant evolves in the saccular phase only between the 19^th^ gestational day and term (day 21–22) [43]. In accordance, our observed surfactant protein A-D mRNA expression and phospholipid levels at E19 were very low, which may have induced a detection error of a true difference. However, at E22, we still found no difference between VD_L_ pups and control pups. The observed lack of impact of vitamin D on these outcomes does not rule out that at higher s-25(OH)D concentrations in the saccular phase can have effects as observed by others [30, 33].

We further described the immunohistochemical localization and relative expression of VDR in the preterm pup and term pup lung. Previous studies have shown that the widely used antibody 9A7γ [32–34] not only binds to VDR, but also possesses non-specific interactions with yet unidentified proteins [44, 45]. Based on a parallel comparison of a large selection of VDR antibodies [44], we chose to use the mouse monoclonal antibody D-6, which possesses the highest specificity, sensitivity and versatility. Our study brings further evidence that the VDR is present in the ATII cells during the last period of gestation, but with unaffected expression by s-25(OH)D in the studied concentration range. Furthermore, our study showed a significantly higher expression of VDR in the E19 pups compared to E22 confirming previous observations of reduced VDR expression in the last days before term in rat offspring [34, 36].

Despite these null-findings for the surfactant synthesis and VDR expression, premature pups with the lowest s-25(OH)D levels had decreased oxygenation and survival time. In humans, the postmortem diagnosis of pulmonary hypoplasia is based on a low LW/BW ratio [46], and we found a strong negative correlation between LW/BW ratio and oxygenation in the premature pups. The premature VD_L_ pups had both significantly reduced lung weight and LW/BW ratio, supporting that pulmonary hypoplasia was likely to be responsible for the decreased survival. The significant positive correlation between birth weight and oxygenation may further indicate a reduced muscle mass leading to earlier muscular fatigue. Moreover, muscular weakness associated to the well-known vitamin D deficiency-associated myopathy in both humans and rodents [47] and lower levels of s-total calcium may have been a contributing factor. We were, however, not able to observe differential signs of muscular fatigue, or increased respiratory distress with increased respiratory muscular work between the pup groups. E19 pups hardly moved and showed only very little respiratory effort at all and E22 pups showed no visual signs of muscular fatigue or respiratory distress. Decreased mineralization and hence more soft ribs as a further explanatory factor was not supported by our whole-body DXA-scans, which showed unchanged bone mineral content between vitamin D depleted offspring and controls at both E19 and E22 (data not shown). This finding is in accordance with findings by Zosky *et al*. [25].

The significantly reduced BW in the premature VD_L_ pups may indicate a more general affection of organ development *in utero* as a result of the severe vitamin D deficiency as previously shown [3, 13, 48, 49]. In humans, a high PW/BW ratio has been associated with RDS, low Apgar scores and increased risk of admission to the neonatal intensive care unit [21, 23]. We showed that the reduced birth weight was not secondary to a reduced placental weight, which was unchanged. Although there are conflicting results in both animal and human data assessing the role of vitamin D in somatic growth [25, 48, 50, 51], there are several biological mechanisms possibly connecting maternal vitamin D to fetal growth.

The vitamin D conversion enzyme gene, *CYP27B1*, and *VDR* are expressed in both placenta and lungs [50, 52]. In placenta, 1,25(OH)_2_D stimulates the secretion of placental hormones, which supports fetal growth and energy needs through delivery of calcium as well as glucose and fatty acids [17, 50]. In the lungs, 1,25(OH)_2_D stimulates pulmonary artery endothelial cell (PAEC) growth in the same manner as vascular endothelial growth factor (VEGF) [52]. Furthermore, 1,25(OH)_2_D regulates transforming growth factor β1 (TGF-β1), a known mediator of airway remodeling [53, 54].

The observed lack of differences in pulmonary and survival outcomes between dietary groups in mature pups may have several explanations: The VDR expression in the lungs was lower at E22 compared to E19 suggesting that pulmonary vitamin D effects are of minor importance at term compared to the preterm period. Moreover, term offspring do not suffer from respiratory insufficiency, why an effect on oxygenation and survival-rate should not be suspected in the mature lung.

We observed reduced CRL in the VD_L_ pups compared to control pups at E22, but not at E19. Direct association analyses across dietary groups between maternal s-25(OH)D and CRL at E22, but not at E19, supported these findings. Vitamin D deficiency *per se*, as well as *VDR* mutations and *VDR* knock-out, has shown not to affect serum mineral concentrations, bone mineralization and fetal longitudinal bone growth [55, 56]. Moreover, animal studies in severely vitamin D deficient rats [57–60] and *VDR*-null mice [61, 62] showed a normal increase in intestinal calcium absorption during pregnancy and normal mineral content of the term offspring. Thus, a low calcium effect on bone growth could not readily explain the significant VD_L_ dependent shortening of CRL at E22. However, CRL is a measure of skull and spine growth rather than longitudinal bone growth and bone mineralization and our association analyses showed not only increased CRL, but also BW and PW at E22 with higher s-25(OH)D, suggesting a general growth-promoting effect of vitamin D at term.

Strengths of the present study include the randomized, blinded design, the obtained severe deficiency level of s-25(OH)D in the VD_L_ group, the between-group similarity in maternal weight gain, duration of anesthesia and maternal SaO_2_ during cesarean section. The difference in s-25-(OH)D between the dietary groups, was further designed to mimic differences in vitamin D status in the clinical setting rather than extreme vitamin D depletion versus pharmacological supplementation.

Limitations included the large with-in group variation resulting in overlapping s-25(OH)D concentrations between the dietary groups. Yet, s-25(OH)D was associated with pup anthropometrics, SaO_2_ and survival time in our *post hoc* analyses, which supported the findings between the dietary groups. The females were ten weeks old and may not have been housed under conditions similar to ours, in terms of lighting and diet, before purchase potentially causing variation in s-25(OH)D which have a long half-life of 2–3 weeks [63, 64]. Variations in appetite may also have contributed to the relatively high within-group variation. To reduce the within group variations and ensure a larger difference between the dietary groups, future studies should assign the diets to young females immediately after the weaning from their mothers. We did not measure pup s-25(OH)D, because of technical volume limitations. However, maternal s-25(OH)D correlates strongly to offspring s-25(OH)D concentrations [65]. Lastly, the fragility of the lung tissue, especially at E19, did not allow *in situ* fixation of the premature lungs under physiological pressure enabling comparison of lung morphology between groups.

The perspectives of our study include the emphasis of whole body animal models to study the effects of vitamin D on the lungs. Our results indicate that aggravation of respiratory failure may occur due to reduced lung and birth weight as the result of severe vitamin D depletion. However, we were unable to show a direct effect of vitamin D deficiency on surfactant measures or VDR expression. Future studies should pursue to evaluate our results using an optimized version of our model with animals assigned to the diets immediately after weaning.

In conclusion, vitamin D depletion during pregnancy led to a lower SaO_2_ and shorter survival-rate in premature rat offspring despite no reduction in lung surfactant constituents. Explanatory factors include reduced lung weight, which may imply a reduced total lung diffusion area, and decreased birth weight, which may indicate a reduced muscle mass leading to earlier muscular fatigue. In support of the hypothesis, vitamin D alleviates respiratory insufficiency at preterm birth. Studies of vitamin D effects in human preterm neonates regarding respiratory insufficiency are warranted.

## Supporting Information

S1 Table (DOCX)

## Abbreviations

CRL: crown-rump length
LW/BW ratio: lung weight/birth weight ratio
PW/BW ratio: placental weight/birth weight ratio
S-25(OH)D: serum 25-hydroxyvitamin D
SaO_2_: oxygen saturation
VD_L_: low vitamin D diet
VDR: vitamin D receptor
