# Supplementary material for: Vitamin D Depletion in Pregnancy Decreases Survival Time, Oxygen Saturation, Lung Weight and Body Weight in Preterm Rat Offspring
Source: PLoS One. 2016 Aug 29;11(8):e0155203. doi: 10.1371/journal.pone.0155203 (PMC5003352; doi:10.1371/journal.pone.0155203)
Supplement: S1 Table — (DOCX) [file pone.0155203.s001.docx]

**S1 Table.**

|  | **E19** | | | **E22** | | |
| --- | --- | --- | --- | --- | --- | --- |
|  | **VD_L_ group (n=9)** | **Controls (n=8)** | **p-value^§^** | **VD_L_ group (n=9)** | **Controls (n=8)** | **p-value^§^** |
| S-25(OH)D before mating (nmol/L) ^*^ | 42 (24-130) | 61 (57-69) | 0.357 | 43 (34-94) | 67 (42-118) | 0.029 |
| S-25(OH)D after CS (nmol/L) ^**^ | 13 (8-27) | 25 (24-66) | 0.002 | 11 (8-17) | 53 (17-137) | 0.001 |
| S-total calcium (nmol/L) before mating ^*^ | 2.61 ± 0.05 | 2.59 ± 0.02 | 0.746 | 2.67 ± 0.04 | 2.64 ± 0.02 | 0.512 |
| S-total calcium (nmol/L) after CS^**^ | 2.70 ± 0.02 | 2.94 ± 0.09 | 0.014 | 2.76 ± 0.08 | 2.79 ± 0.05 | 0.777 |
| S-phosphate (nmol/L) before mating ^*^ | 2.60 ± 0.12 | 2.78 ± 0.17 | 0.391 | 2.56 ± 0.09 | 2.93 ± 0.27 | 0.194 |
| S-phosphate (nmol/L) after CS^**^ | 1.81 ± 0.07 | 2.15 ± 0.12 | 0.023 | 1.87 ± 0.08 | 1.93 ± 0.14 | 0.705 |

^*^Tail vein. ^**^Intracardial puncture. ^§^No correction for multiple comparisons. Normal distributed data presented as mean ± SEM. Non-normal distributed data presented as median (range). Abbreviations: S-25(OH)D: serum 25-hydroxyvitamin D, CS: cesarean section.
